# Formation of a Unique Cluster of G-Quadruplex Structures in the HIV-1 *nef* Coding Region: Implications for Antiviral Activity

**DOI:** 10.1371/journal.pone.0073121

**Published:** 2013-08-27

**Authors:** Rosalba Perrone, Matteo Nadai, Jerrod A. Poe, Ilaria Frasson, Manlio Palumbo, Giorgio Palù, Thomas E. Smithgall, Sara N. Richter

**Affiliations:** 1 Department of Molecular Medicine, University of Padua, Padua, Italy; 2 Department of Microbiology and Molecular Genetics, University of Pittsburgh School of Medicine, Pittsburgh, Pennsylvania, United States of America; 3 Department of Pharmaceutical and Pharmacological Sciences, University of Padua, Padua, Italy; University of Kansas Medical Center, United States of America

## Abstract

G-quadruplexes are tetraplex structures of nucleic acids that can form in G-rich sequences. Their presence and functional role have been established in telomeres, oncogene promoters and coding regions of the human chromosome. In particular, they have been proposed to be directly involved in gene regulation at the level of transcription. Because the HIV-1 Nef protein is a fundamental factor for efficient viral replication, infectivity and pathogenesis *in* ** *vitro* and *in* ** *vivo*, we investigated G-quadruplex formation in the HIV-1 *nef* gene to assess the potential for viral inhibition through G-quadruplex stabilization. A comprehensive computational analysis of the *nef* coding region of available strains showed the presence of three conserved sequences that were uniquely clustered. Biophysical testing proved that G-quadruplex conformations were efficiently stabilized or induced by G-quadruplex ligands in all three sequences. Upon incubation with a G-quadruplex ligand, Nef expression was reduced in a reporter gene assay and Nef-dependent enhancement of HIV-1 infectivity was significantly repressed in an antiviral assay. These data constitute the first evidence of the possibility to regulate HIV-1 gene expression and infectivity through G-quadruplex targeting and therefore open a new avenue for viral treatment.

## Introduction

G-quadruplexes (G-4s) are inter- and intramolecular four-stranded structures formed by G-rich DNA and RNA. G-4s are based on the formation of G-quartets, which are stabilized by Hoogsteen-type hydrogen bonds between guanines and by the interaction with cations located between the tetrads. G-quartets stack on top of each other to give rise to G-4s. G-rich oligonucleotides can be very polymorphic, and the adopted structures are dependent on several factors, including the base sequence, strand concentration, loop connectivities, and cations present. Such tetraplex DNAs have been an emerging topic in nucleic acids research because of the recent indication of their involvement in a series of key biological functions.

Initially G-4s have been shown to form *in* ** *vitro* [1] and *in* ** *vivo* [2] in the G-rich sequence of telomeres: stabilization of the G-4 folded structure has been proposed as an effective approach to inhibit telomerase activity in tumour cells [3]. Subsequently, it has been shown that G-rich sequences are tightly clustered immediately upstream and downstream of the transcription start site in proliferation-associated genes, therefore suggesting a role of tetraplexes as *c* *i* *s*-acting regulatory elements in gene expression [4], in particular in genes important in cell signalling, which have representatives from the six hallmarks of cancer [5].

G-rich sequences capable of forming G-4 were also found within coding regions of the human genome, including minisatellites [6], immunoglobin heavy chain switch regions [7] and rDNA [8], and were shown to be the target of binding proteins [9]. The folding of DNA G-4 most likely occurs during single-stranded DNA formation, i.e. during transcription and replication [10]. Recent work has shown that G-4 can also form within coding regions: on the leading strand G-4s arise during replication and promote genetic instability in human and yeast [11,12]; on the lagging strand G-4-forming sequences were found to generate G loops during transcription both *in* ** *vitro* and in *Escherichia coli* [13,14]. Further, association between G-4-single nucleotide polymorphisms and expression of the corresponding gene in individuals has been proposed [15]. Recently, cell-cycle dependent G-4 formation in living cells and their stabilization by G-4 ligands has been demonstrated [16].

To date, a diverse array of G-4 stabilizing compounds have been identified. General features of these G4-recognising ligands include a large flat aromatic surface and cationic charges [17]. Examples include perylenes, such as PIPER [18], porphyrins, such as TMPyP4 [19], trisubstituted acridines, such as BRACO-19 [20], natural macrocycles, such as Telomestatin [21], and fluoroquinolone derivatives, such as Quarfloxin [22]. Some of these compounds have shown encouraging anticancer activity *in* ** *vitro*, *in* ** *vivo* and in clinical trials [17,22].

The importance of G-4-forming sequences as regulatory systems has been so far demonstrated only in eukaryotic cells, however the presence of G-4 sequences has been recently pinpointed also in prokaryotic cells [23–25]. Similarly, it is likely that other organisms, such as viruses, have evolved analogous regulatory mechanisms. Nevertheless, research in this area has been so far very elusive.

The human immunodeficiency virus (HIV) is the etiological agent of the acquired-immuno-deficiency syndrome (AIDS). HIV establishes a persistent infection in human hosts, with the depletion of CD4+ lymphocytes, the major target cells of viral infection *in* ** *vivo*, eventually resulting in defective cellular immunity, and thus leading to full-blown AIDS [26]. HIV is a member of the genus *Lentivirus*, part of the family of *Retroviridae*. Two types of HIV have been characterized: HIV-1 and HIV-2. The strains of HIV-1 can be classified into four groups: the "major" group M, the "outlier" group O and two new groups, N and P. More than 90% of HIV-1 infections belong to HIV-1 group M [27]: within this group, at least nine genetically distinct subtypes or clades (A, B, C, D, F, G, H, J and K) of HIV-1 have been reported [28]. The viral genome consists of two copies of positive single-stranded RNA that codes for nine genes (*gag*, *pol*, *env*, *tat*, *rev*, *vif*, *vpr*, *vpu*, *nef*) [29].

To date, FDA-approved antiretroviral drugs belong to several classes, based on their mechanism of action: 1) nucleoside and 2) non-nucleoside RT inhibitors; 3) protease, 4) fusion and 5) integrase inhibitors and 6) CCR5 antagonists [27]. Combinations of the existing drugs are very effective in slowing down progression to AIDS; however, the high mutation rate of HIV gives rise to resistance which ultimately impairs antiretroviral therapy. Therefore, there is an urgent need for new anti-HIV drugs with an innovative mechanism of action, possibly against highly conserved viral sites.

One attractive target for the anti-HIV therapy is the Nef protein. Nef is a small myristoylated protein expressed early in the HIV-1 life cycle: it is a fundamental factor for efficient viral replication and pathogenesis *in* ** *vivo*; it also facilitates virus replication and enhances viral infectivity *in* ** *vitro* [30–33]. An essential role for Nef *in* ** *vivo* has been demonstrated in a subset of long-term non-progressors, HIV-infected individuals that do not progress to AIDS. Viral isolates from some of these individuals exhibit either a deletion in the *nef* gene or defective *nef* alleles [34]. In addition, rhesus macaques infected with an engineered strain of SIV that lacked the functional Nef protein also did not attain high viral loads and did not progress to clinical disease [35]. Nef alters host cell processes by several mechanisms: for example, to promote escape from the immune system and infectivity, it downregulates CD4 and MHC I expression on the cell surface, to enhance viral replication and infectivity it activates CD4+ CTL and downregulates/interacts with several cellular factors [36]. The Nef coding region, a 621 bp-long sequence located at the 3’-end of the viral genome, partially overlaps with the 3’-long terminal region (LTR).

The presence of G-4 forming sequences in the *nef* gene has never been reported. At the viral DNA level, G-4 forming sequences have only been described in a single-stranded portion (central DNA flap) of the reverse-transcribed pre-integration HIV-1 genome, which specifically interacts with the viral nucleocapsid protein and protects the pre-integrated genome from nuclease degradation [37]. In the HIV-1 RNA genome, a G-4 structure has been proposed to promote dimerization of the two viral genome copies [38].

We present here the first comprehensive analysis of putative G-4 forming sequences in the HIV-1 *nef* coding region. We showed that three contiguous putative G-4 regions are present and that at least two are extremely conserved among most circulating HIV-1 strains. We provide evidence of their G-4 folding and stabilization in the presence of cations and G-4 binding compounds by spectroscopic and electrophoretic techniques. Finally, we demonstrate that G-4 ligands impaired Nef expression and significantly suppressed Nef-dependent enhancement of HIV-1 infectivity.

## Materials and Methods

All oligonucleotides were purchased from Sigma-Aldrich (Milan, Italy). TMPyP4 and PIPER were purchased from Calbiochem, (Merck Chemicals Ltd, Nottingham, UK). BRACO-19 was obtained from ENDOTHERM GmbH, (Saarbruecken, Germany).

### G-4 analysis of the HIV-1 genome

The HIV-1 Nef coding region (strain HXB2/LAI, NC_001802) was analyzed by QGRS Mapper (http://bioinformatics.ramapo.edu/QGRS/index.php) for prediction of G-4 forming sequences in both coding and non-coding strands [39]. The putative G-4s were identified by the motif G_x_N_y1_G_x_N_y2_G_x_N_y3_G_x_, where x was the number of guanine (G) tetrads and y_n_ the length of loops connecting the G tetrads. The following restrictions have been applied: i) the number of tetrads had to be ≥ 2; ii) maximum length of QGRS was set to 30 bases; iii) at most one of the loops was allowed to be of zero length; iv) loop size from 0 to 15. The found QGRS were ranked based on the G-score, which is the likelihood to form a stable G-4, according to the following principles: a) shorter loops are more common than longer loops; b) G-4s tend to have loops roughly equal in size; c) the greater the number of G tetrads, the more stable the G-4.

Three G-4 putative sequences (Nef 8528 and Nef 8624 in coding strand, Nef 8547 in non-coding strand) were evaluated for their consensus sequence by aligning 3224 Nef sequences (HIV-1, M Group) from the HIV database (http://www.hiv.lanl.gov/content/sequence/NEWALIGN/align.html) using Jalview (http://www.jalview.org/). Aligned sequences were searched for patterns expressed as Perl compatible regular expressions using GNU grep command line tool.

### Circular dichroism and UV spectroscopy

DNA oligonucleotides used for spectral analysis were Nef8528 5’-GAGGAGGAGGTGGGT-3’, Nef8547 5’-GGTCTTAAAGGTACCTGAGGTCTGACTGG-3’, Nef8624 5’-GGGGGGACTGGAAGGG-3’ and their complementary sequences, Nef8528-Compl ACCCACCTCCTCCTC, Nef8547-Compl CCAGTCAGACCTCAGGTACCTTTAAGACC, Nef8624-Compl CCCTTCCAGTCCCCCC. All oligonucleotides were diluted from stock to the final concentration (4 µM) in lithium cacodylate buffer (10 mM, pH 7.4) and, where appropriate, KCl 100 mM. All samples were annealed by heating at 95 °C for 5 min, gradually cooled to room temperature and measured after 24 h. Compounds at 16 µM final concentration were added after DNA annealing. CD spectra were recorded on a Jasco-810 spectropolarimeter (Jasco, Easton, MD, USA) equipped with a Peltier temperature controller using a quartz cell of 5-mm optical path length and an instrument scanning speed of 100 nm/min with a response time of 4s over a wavelength range of 230-600 nm. The reported spectrum of each sample represents the average of 2 scans at 20°C and is baseline-corrected for signal contributions due to the buffer. Observed ellipticities were converted to mean residue ellipticity (θ) = deg × cm^2^ × dmol^−1^ (mol. ellip.). For the determination of T_m_, spectra were recorded over a temperature range of 20-95 °C, with temperature increase of 5 °C/min. T_m_ values were calculated as the first derivative of the melting profiles. For UV thermal unfolding, DNA oligonucleotides were diluted to final concentration of 4 µM or 40 µM. UV spectra were recorded on Lamba25 UV/Vis spectrometer (PerkinElmer) equipped with a Peltier temperature controller using a quartz cell of 10-mm optical path length and measuring absorbance at 295 nm. UV spectra were recorded over a temperature range of 20-95°C, with temperature increase of 1°C/min. The autozero function was applied on the corresponding buffer sample at 20°C.

### Taq polymerase stop assay

DNA templates bearing the sequences of interest with G possibly involved in G-4 formation (in bold) and the primer annealing region (underlined) were: Nef8528pol 5’-TT**G** **G**A**G** **G**A**G** **G**T**G** **G** **G**TTTTC C A G T C A C A C A C C T C A G-3′, Nef8624pol 5’-TT**G** **G** **G** **G** **G** **G**ACT**G** **G**AA**G** **G** **G**TTTTC C A G T C A C A C A C C T C A G-3’, Nef8547pol 5’-TT**G** **G**TCTTAAA**G** **G**TACCTGA**G** **G**TCTGACT**G** **G**TTTTC G A G A C A C A G C T C A G-3’. DNA templates bearing control sequences were: (NefControl1 5’-TTGTCGTCACAGTCTGACTGTTTT C C A G T C A C A C A C C T C A G-3′ and NefControl2 5’- TTGTCGTTGAAGATAGCCGTGTAGCTGACGTTTTTC G A G A C A C A G C T C A G-3’). DNA primers were: 5′-ATCGATCGCTTCTCGTC T G A G G T G T G T G A C T G G-3′ and 5’-ATCGATCGCTTCTCGTC T G A G C T G T G T C T C G-3’, where the underlined nucleotides are those complementary to the templates. Primers were 5′-end-labeled with [γ-^32^P] ATP using T4 polynucleotide kinase for 30 min at 37°C and purified with Illustra MicroSpin G-25 Column (GE Healthcare, Life Sciences, Milan, Italy).

DNA templates were diluted from stock to the final concentration (50 µM) in lithium cacodylate buffer (10 mM, pH 7.4) with 100 mM KCl and then let fold by heating at 95 °C for 3 min, gradually cooled to room temperature, and incubated at 4 °C overnight. DNA templates were further diluted to a concentration of 1 µM and mixed with DNA primer (200 nM), 1X PCR reaction buffer (Applied Biosystems, Carlsbad, California, USA), and 0.1 mM dNTPs. Where appropriate, TMPyP4 was added. AmpliTaq Gold DNA polymerase (1U/reaction, Applied Biosystem, Carlsbad, California, USA) was then added. Samples were subjected to 30 cycles: 95°C 30 sec, 60°C 30 sec, 72°C 30 sec. Reactions were stopped by ethanol precipitation. Marker lanes were generated on the labelled double stranded PCR product using the Maxam & Gilbert protocol. Briefly, ethanol precipitated PCR products were treated with formic acid (12 µl) for 5 min at 20°C. Reactions were stopped by ethanol precipitation. Samples were treated with piperidine 1 M for 30 min at 90°C; reactions were stopped on ice for 5 min. Samples were concentrated in SpeedVac. Markers corresponded to the C-rich complementary strand. The primer extension products and markers were separated on a 12% polyacrylamide denaturing gel and visualized by phosphorimaging (Typhoon FLA 9000, GE Healthcare, Milan, Italy).

### Cloning

Plasmid p-Nef-HA EGFP-N1 containing Nef-HA fused to GFP was obtained by PCR amplification of the Nef-HA coding sequence (strain HXB2/LAI; NC_001802) in plasmid pNefHABJ5 (kindly donated by Prof. M. Pizzato, Centre for Integrative Biology, University of Trento, Trento, Italy). PCR was performed using primers prFNef (5’-TAAGCTAGCACGCGTCATGGGTGGCAAGTGG-3’) and prRNef (5’-ACTGAATTCTAGCGTAATCTGGGACGTC-3’), which introduced NheI and EcoRI restriction sites for subsequent insertion in the pEGFP-N1 vector (Clontech, Mountain View, CA, USA). The obtained coding sequence of the fused Nef-HA GFP protein was confirmed by sequencing.

### Flow cytometry analysis

For FACS analysis, 8 × 10^4^ of HEK 293T cells were seeded in a 12-well plate in 1 ml of DMEM/10% FBS medium and incubated for 24 hours. Cells were next transfected with pNef-HA EGFP-N1 by *T* *r* *a* *n* *s*IT-293 Transfection Reagent (Mirus, Madison, WI, USA). After 4 hours, cells were treated with TMPyP4 or TMPyP2 (10 µM) and incubated overnight. After trypsinization, cells were washed with PBS and resuspended in 500 µl of PBS. To evaluate GFP expression, a total of 30000 events were acquired for each sample with an LRS 2 instrument using FACS DIVA Software (BD Bioscience, San Jose, CA, USA) and analyzed with FlowJo (Tree Star, OR, USA).

### Cytotoxicity assays

Evaluation of the cytotoxicity of the compounds in HEK 293T cells was performed using a 3-(4,5-dimethylthiazol-2-yl)-2,5-diphenyltetrazolium bromide (MTT) assay. HEK 293T cells were seeded in 96-well plate (1,5 x 10^4^/well) and grown overnight. Test compounds were added to one series of triplicate wells and incubated at 37°C. After 48 h, MTT (Sigma-Aldrich, Milan, Italy) solution (5mg/ml in PBS) was added and incubated at 37°C. After 4h, a solubilizing solution (SDS 10%, HCl 10mM) was added to dissolve the formazan crystals and incubated overnight at 37°C. The blue-purple formazan corresponding to metabolically active cells was then measured spectrophotometrically. The absorbances at 620nm were read in a computer-controlled spectrophotometer with a 96-well plate reader (Sunrise, Tecan) using the program Magellan 4.0. The percentage of cell viability was calculated by using the median absorbance value of three wells compared to the control’s wells. CC_50_ was the concentration of the test compounds that caused death of 50% cell population.

TZM-bl cells [40,41] were seeded in 96-well plates (2 x 10^4^) and grown overnight to permit adherence. Cells were then incubated with compounds in DMSO carrier solvent and incubated at 37°C. After 48 h, cytotoxicity was assessed using the Cell Titer Blue reagent (Promega) and the manufacturer’s protocol.

### Antiviral assays

Viral stocks were prepared by transfection of HEK 293T cells (ATCC) with wild-type and Nef-defective (ΔNef) proviral genomes (NL4-3 strain) and amplified in the T cell line, MT2 (NIH AIDS Research and Reference Reagent Program) as previously described [42–44]. HIV-1 infectivity was measured using the TZM-bl reporter cell line [40,41] (NIH AIDS Research and Reference Reagent Program). TZM-bl cells support HIV-1 replication in a Nef-dependent manner and contain a luciferase reporter under the control of the HIV-1 promoter [41,45]. Cells were seeded in 96-well plates (2 x 10^4^) and grown overnight to permit adherence prior to treatment and viral infection. TMPyP4 was solubilized in DMSO and preincubated separately with both the cell culture medium (100 µL) and wild-type HIV-1 or ΔNef HIV-1 (100 µL) for 4 h prior to infection in a combined final volume of 200 µL. After 48 h at 37°C, the cells were washed with PBS and lysed in luciferase lysis buffer (Promega) by rocking for 15 min. Lysates (40 µL) were transferred to white 96-well plates, and 50 µL luciferase reagent (Promega) was injected into each well. Readings were recorded with a delay time of 2 s and an integration period of 10 s.

## Results

### Computational analysis of the HIV-1 Nef coding region for the presence of putative G-4 forming regions

The HIV-1 Nef coding region (strain HXB2/LAI, NC_001802) was analysed with QGRS Mapper which is an online algorithms-based software program for recognition and mapping of putative Quadruplex forming G-Rich Sequences (QGRS) [39]. The found QGRS were ranked based on the G-score, which is the likelihood to form a stable G-4.

Since the viral genome is retrotranscribed into double-stranded DNA and inserted into the human genome, analysis for QGRS was performed also on the reverse strand of the *nef* coding gene.

**Figure 1:**
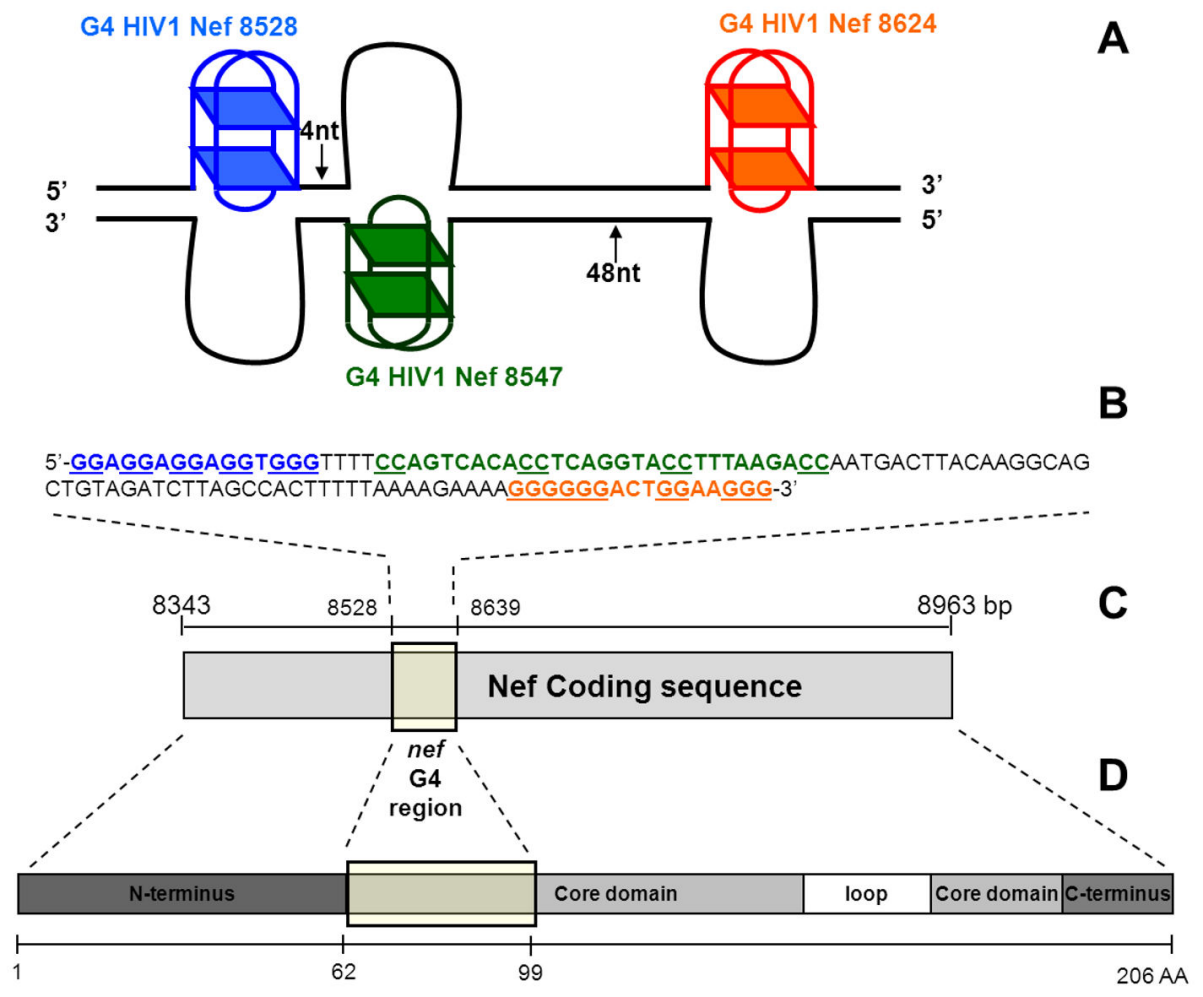
Putative G-forming regions in the HIV-1 *nef* coding region. A) Scheme of G-4 formation within the double-stranded DNA of the *nef* region: Nef8528, Nef8547, Nef8624 G-4 structures are shown in blue, green and red, respectively. The numbers of nts separating each G-4 structure are indicated. The scheme indicates the possibility of formation of a cluster of non-canonical DNA structures within a small portion (112 nts) of the HIV-1 genome. B) Nucleotide sequence of the *nef* coding region where three putative G-4 sequences were identified. Nef8528 is shown in blue and Nef8624 in red. Nef8547 was identified on the non-coding strand, thus the reverse complementary sequence is shown on the upper strand (in green). C) Scheme of the HIV-1 nef coding sequence with numbering referring to the HIV-1 strain HXB2/LAI, NC_001802. D) Scheme of the aminoacidic sequence of the Nef protein indicating reported structural domains [73]. The protein moiety coded by the G-4 rich nucleotide region is highlighted by the rectangular yellow shape, indicating involvement of the conserved N-terminal Nef core region. Note that the first three nts of the Nef8528 sequence exactly code for the first amino acid of the protein core region.

Four putative QGRS were located in the *nef* gene (Table 1): two on the forward (positions 8528 and 8624, where +1 is the first base of the HIV-1 genome as reported in GenBank, NC_001802) and two on the reverse strand (positions 8547 and 8727). In particular, the three that displayed the highest G-score (≥ 20) were also adjacent to one another: the first on the forward strand (Nef8528) and the second on the reverse strand (Nef8547) were separated by just 4 nucleotides (nts); the second from the third sequence on the forward strand (Nef8624) by 48 nts (Figure 1A-B). If these three sequences were able to fold in G-4, this region of the HIV-1 proviral genome could constitute an important cluster of non-canonical DNA structures with possible effects on polymerase processing (and therefore impact on replication and transcription events) (Figure 1C-D). It is interesting to note that all selected G-4 putative sequences code for amino acids of the Nef core, which is the most conserved region of the protein and is essential for interaction with cellular proteins to mediate key viral functions [46–48] (Figure 1D).

**Table 1:** Putative G-quadruplex forming sequences within the HIV-1 nef gene (HIV-1 strain HXB2/LAI, NC_001802) in the forward and reverse strand.

**Position** **** **in** **** **the** **** **F** **** **strand**	**Length**	**Putative** **** **G-4** **** **nef** **** **sequence**	**G-score**
8528	15	**G** **G**A**G** **G**A**G** **G**A**G** **G**T**G** **G** **G**	17-21
8547	29	**C** **C**AGTCACA**C** **C**TCAGGTA**C** **C**TTTAAGA**C** **C**	21
8624	16	**G** **G** **G** **G** **G** **G**ACT**G** **G**AAG**G** **G**	16-20
8727	30	**C** **C**AGGG**C** **C**AGGGGTCAGATAT**C** **C**ACTGA**C** **C**	12

To establish the importance of the identified sequences from a virus standpoint, the degree of conservation in terms of sequence and G-4 formation among HIV-1 strains was assessed.

Initially, the presence of the exact sequences identified in the HIV-1 HXB2/LAI strain was analysed in 3224 *nef* sequences of the HIV-1 M group reported in the HIV database (http://www.hiv.lanl.gov/content/sequence/NEWALIGN/align.html). Among these, 1538 sequences belonged to clade B, 612 to clade C, 486 to clade A, and 588 to other clades. As shown in Table 2, Nef8528, Nef8547 and Nef8624 were fairly well conserved in the M group, especially in the B subtype, where conservation was higher with respect to the other clades of the M group. Next, the possibility of G-4 formation was statistically analysed by maintaining the number and size of G repeats, while varying loop regions. Two cases were considered: in instance i) loops could diverge in base composition while maintaining a constant length; in instance ii) both loop composition and length were allowed to vary. As shown in Table 2, Nef8547 and Nef8624 reached a degree of conservation higher than 95% in both cases across all considered HIV subtypes. Nef8528 was conserved to a significant extent in clades A and B (up to 66.3%); its presence was negligible only in clade C. Consensus sequences and base conservation in each position are reported in Table 3. Overall these data indicate that the G-4 pattern, therefore the possibility of G-4 folding, in the selected G-rich sequences in the Nef coding region is extremely conserved among circulating HIV-1 strains, at least for Nef8547 and Nef8624.

**Table 2:** Statistical analysis of the conservation grade of the G-4 *nef* sequences or their G-4 patterns.

**G-4** **** **name**	**G-4** **** **sequence** **** **or** **** **pattern**	**Conservation** **** **grade** **** **(** **%)**			
		Group M	Clade A	Clade B	Clade C
Nef8528	**G** **G**A**G** **G**A**G** **G**T**G** **G** **G**	13.9%	0.6%	27.2%	0.3%
A)	G_2_ X_1_ G_2_ X_1_ G_2_ X_1-2_ G_2_	15.7%	2.4%	29.9%	0.5%
B)	G_2_ X_0-7_ G_2_ X_0-7_ G_2_ X_0-7_ G_2_	46.8%	57.6%	66.3%	9.3%
Nef8547	**C** **C**AGTCAGA**C** **C**TCAGGTA**C** **C**TTTAAGA**C** **C**	24.1%	0.0%	39.2%	11.1%
A)	C_2_ X_7_ C_2_ X_7_ C_2_ X_7_ C_2_	98.6%	97.6%	99.1%	98.5%
B)	C_2_ X_1-10_ C_2_ X_1-10_ C_2_ X_1-10_ C_2_	99.0%	99.4%	99.1%	98.9%
Nef8624	**G** **G**GG**G** **G**ACT**G** **G**AA**G** **G** **G**	66.4%	13.3%	86.5%	58.8%
A)	G_2_ X_2_ G_2_ X_3_ G_2_ X_2-3_ G_2_	98.0%	95.2%	97.9%	98.7%
B)	G_2_ X_0-7_ G_2_ X_0-7_ G_2_ X_0-7_ G_2_	99.9%	99.4%	99.9%	100.0%

In bold bases possibly involved in G-4 folding.

**Table 3:** Consensus sequences and percentages of base conservation at each position.

**Nef8528**	**G**	**G**	A	**G**	**G**	A	**G**	**G**	A	**G**	**G**	T	**G**	**G**	**G**														
Consensus seq	G	G	A	G	G	A	G	G	A	G	G	T	A	G	G														
% consensus	76	96	90	35	94	87	65	99	95	50	99	99	51	99	99														
**Nef8547**	**C**	**C**	A	G	T	C	A	C	A	**C**	**C**	T	C	A	G	G	T	A	**C**	**C**	T	T	T	A	A	G	A	**C**	**C**
Consensus seq	C	C	A	G	T	C	A	C	A	C	C	T	C	A	G	G	T	A	C	C	T	T	T	A	A	G	A	C	C
% consensus	99	99	97	99	99	91	98	85	80	99	99	75	99	99	86	99	99	77	99	99	86	79	98	90	99	99	91	99	99
**Nef8624**	**G**	**G**	**G**	**G**	**G**	**G**	A	C	T	**G**	**G**	A	A	**G**	**G**	**G**													
Consensus seq	G	G	G	G	G	G	A	C	T	G	G	A	A	G	G	G													
% consensus	98	99	99	99	99	99	99	99	99	99	99	99	68	99	99	99													

Bases involved in G-4 folding are shown in bold.

### G-rich sequences in the *nef* region are able to fold in G-4

**Figure 2:**
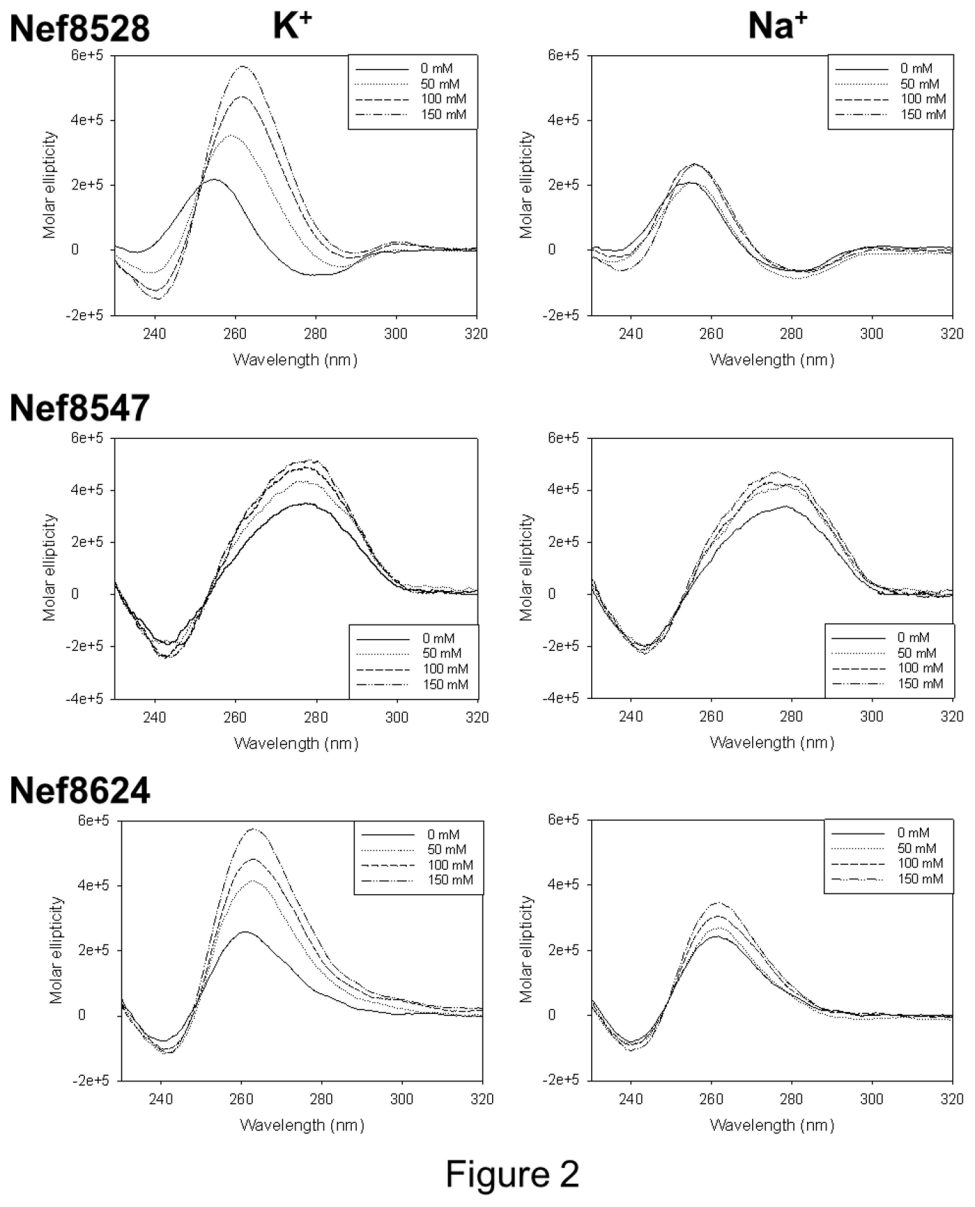
CD spectra of the putative G-4 forming oligonucleotides in the *nef* region. For each oligonucleotide, CD spectra were measured in the absence or presence of increasing concentrations (50-150 mM) of K^+^ or Na^+^ cations.

The actual folding of the selected *nef* sequences in a G-4 conformation was initially assessed by circular dichroism (CD) spectroscopy. In the case of Nef8528, which presents five GG-repeats, we used the minimal sequence that could fold into G-4 (see Materials and Methods). Since monovalent cations, in particular K^+^, are known to stabilize G-4s [49], the three *nef* sequences were incubated in the presence of increasing concentrations of K^+^ or Na^+^. Both cations increased the CD signal, with K^+^ exhibiting a remarkably higher effect than Na^+^. CD analysis is typically used to obtain information on the G-4 topology: in particular, a parallel-type G-4 conformation is manifested by a maximum at 260 nm and a minimum at 240 nm, while an antiparallel topology exhibits two maxima at 290 nm and 240 nm and a minimum at 260 nm [50]. Nef8528 and Nef8624 displayed a clear parallel-type conformation upon addition of K^+^ and Na^+^, with a maximum at 260 nm and a minimum at 240 nm (Figure 2). Conversely, Nef8547 presented a maximum at around 275 nm and a negative peak at 240 nm, with low K^+^/Na^+^ dependence. Therefore, the 7-nt-long loop Nef8547 apparently did not naturally fold into a canonical G-4 conformation: to note, however, that similar CD spectra have been reported for G-4 forming oligonucleotides with long loops (i.e. at least two loops ≥ 5 nts) [51].

**Figure 3:**
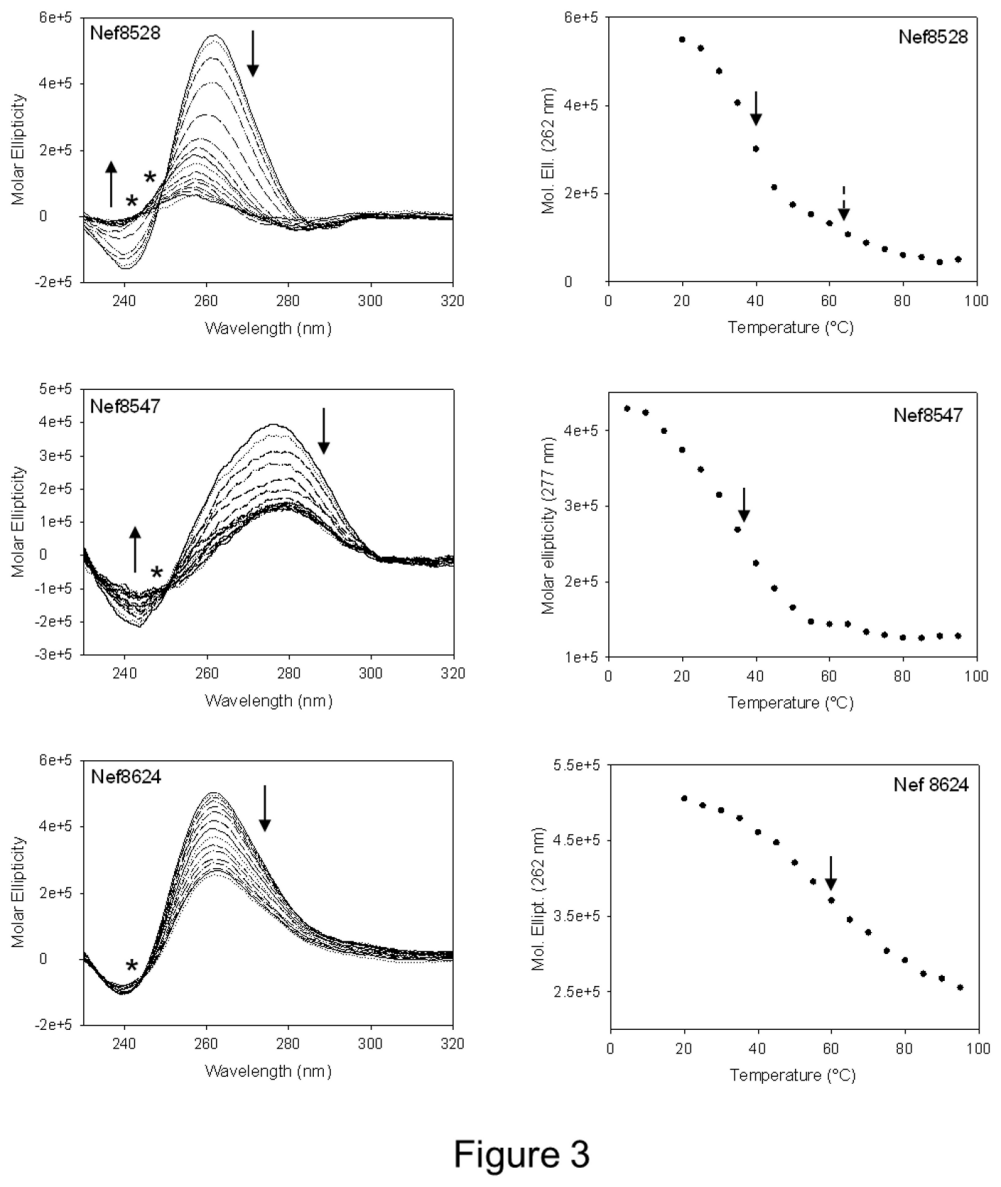
CD thermal unfolding of the G-4 *nef* oligonucleotides. CD spectra measured at increasing temperatures (25-95°C) are shown on the left. Arrows indicate spectral trends at the corresponding wavelengths. Asterisks indicate isosbestic points. Plots of molar ellipticity values (black circles) measured at the indicated wavelength (corresponding to positive peaks) as a function of temperature are reported on the right. Arrows indicate T_m_ points.

Stability of the G-4 structures was next evaluated by CD thermal unfolding and melting temperatures (T_m_) were calculated as the first derivative of the melting profiles (Figure 3 and Table 4). In all cases the CD signal decreased with increasing temperature. For Nef8547 and Nef8624 a single transition between 25°C and 95°C was appreciable leading to discrete T_m_ values (35°C and 59°C, respectively, Table 4). For Nef8528 two transitions were present: a first structural variation at 39°C and a second at T_m_ > 50°C, where a clear inflection point was not observed. This behaviour could also be evinced by spectra overlapping, where two isosbestic points (asterisks in Figure 3) were detected, indicating the presence of at least three spectroscopically distinct species: the initial G-4 structure, a second folded form, likely a more flexible G-4 conformation, and the unfolded random coiled structure. In addition, T_m_ values measured by UV thermal unfolding at oligonucleotide concentration of 40 µM were very similar to those obtained at 4 µM (Table 4), indicating a prevalent intramolecular G-4 folding.

**Table 4:** T_m_ and ΔT_m_ of the three G-4 nef sequences (4 µM and 40 µM) in the absence and presence of G-4 ligands (16 µM) measured by CD and UV spectroscopy.

**G-4** **** **DNA**	**Drug** **** **added**	**Tm** **** **(** **°C** **)** **** **4** **** **µM** *	**ΔTm** **** **(** **°C** **)** **** **4** **** **µM** **	**Tm** **** **(** **°C** **)** **** **40** **** **µM** *
**Nef8528**	**-**	39, > 50	-	41
	**TMPyP4**	57	18	-
	**BRACO**	69	30	-
	**Piper**	80	41	-
**Nef8547**	**-**	35	-	37
	**TMPyP4**	56	21	-
	**BRACO**	48	13	-
	**Piper**	56	21	-
**Nef8624**	**-**	59	-	58
	**TMPyP4**	72	13	-
	**BRACO**	64/75	5/16	-
	**Piper**	61/>100	2/> 40	-

* Average standard deviation was 0.3.

** Average standard deviation was 0.4.

**Figure 4:**
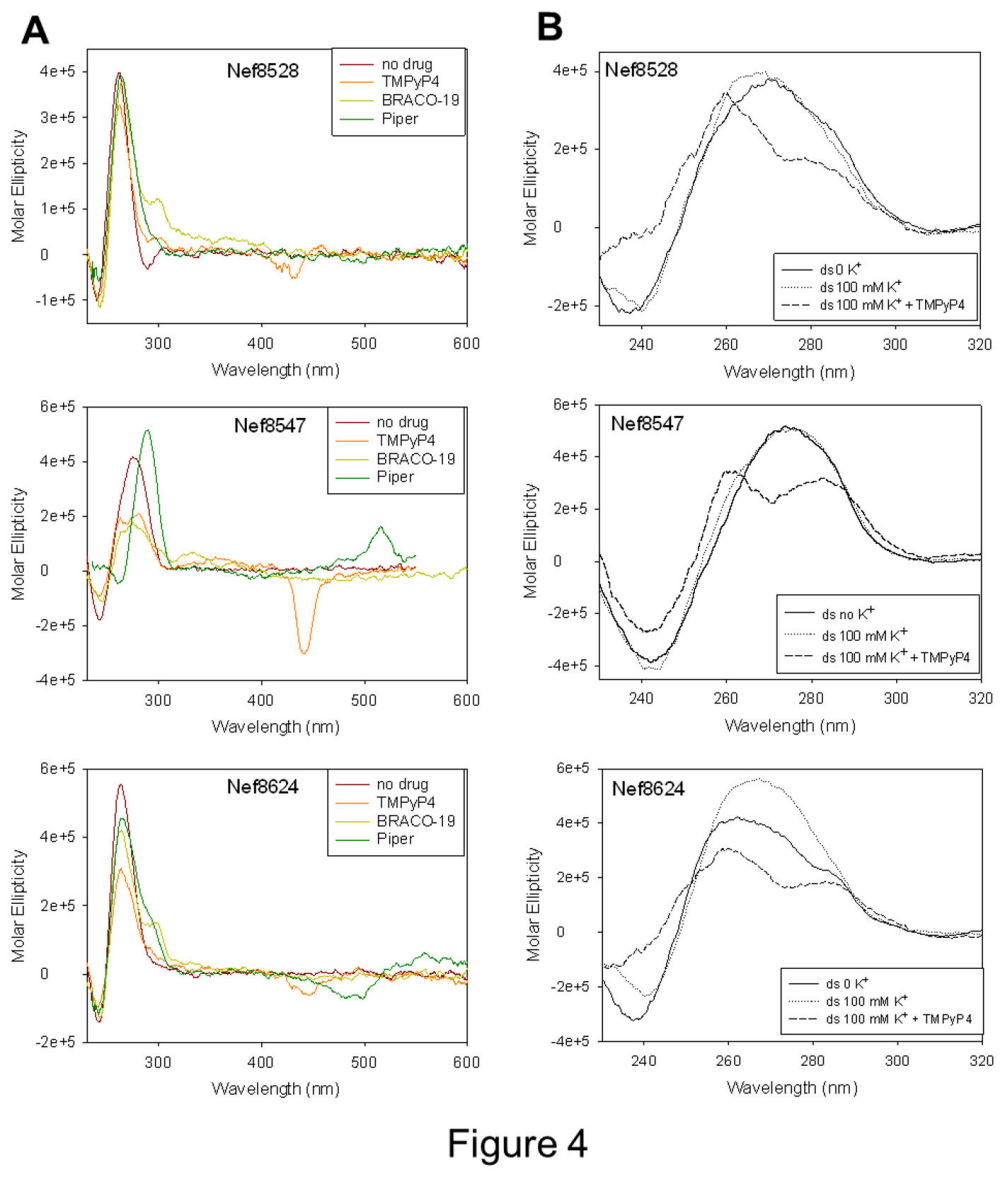
CD spectra of G-4 *nef* single-stranded or double-stranded oligonucleotides in the presence of G-4 ligands. A) CD spectra of G-4 *nef* single-stranded oligonucleotides in the presence of TMPyP4, BRACO-19 or PIPER. Addition of ligands stabilized G-4 conformations and generated ICD bands in the UV/Vis absorption regions of G-4 ligands. B) CD spectra of G-4 *nef* double-stranded oligonucleotides in the presence or absence of K^+^ and TMPyP4. Addition of the G-4 binding compound induced shifting from double-stranded DNA spectra to mixed type G-4 signatures in all three cases.

Several compounds have now been reported to stabilize the G-4 structure. Three G-4 ligands with different central cores (i.e. porphyrin for TMPyP4, acridine for BRACO-19, and perylene for PIPER) were incubated in the presence of each of the three *nef* G-rich oligonucleotides to check for the compound ability to induce/stabilize the G-4 topology. CD thermal unfolding analysis was employed to check stabilization of the G-4 conformation imposed by the G-4 ligands. CD spectra of all three oligonucleotides in the presence of G-4 ligands were characteristic of G-4 conformations. In particular, Nef8528 and Nef8624 maintained the initial parallel-like topology with TMPyP4 and PIPER; in the presence of BRACO-19 an additional positive peak appeared at 290 nm, which is characteristic of a G-quadruplex topology and might depict a shifting towards a hybrid topology. In the case of Nef8547, PIPER induced an antiparallel-like spectrum, while TMPyP4 and BRACO-19 stabilized hybrid-type G-4 structures (Figure 4A), indicating that G-4 ligands are able to drive Nef8547 folding into a G-4 conformation. To note that in most cases an induced CD spectrum was observed in the UV/Vis absorption region of the ligands, further confirming oligonucleotide/compound interaction. G-4 ligands highly stabilized the G-4 conformations of Nef8528 and Nef8547 (Table 4). Nef8624 in general was less efficiently stabilized, likely due to the higher innate stability of this latter oligonucleotide. In cases where two transitions were observed, T_m_ values for each transition were reported (Table 4). These data altogether confirm the ability of the *nef* sequences to fold in G-4: G-4 ligands can bind, induce and stabilize their G-4 conformations.

Since the G-rich *nef* sequences in the proviral genome are normally embedded in a DNA double-helix, TMPyP4 was incubated with each *nef* oligonucleotide in the presence of its complementary counterpart in order to assess the ability of a G-4 ligand to promote G-4 folding from a double-stranded (ds) DNA. CD spectra of the ds oligonucleotides in the absence or in the presence of K^+^ were very similar and characteristic of a B-DNA [52]. However, when TMPyP4 was added, the CD spectra clearly shifted, presenting two maxima at 290 and 260 nm, which are indicative of a G-4 conformation (Figure 4B). Since TMPyP4 absorbance below 300 nm is extremely low [53], the observed molar ellipticity variation must be due to changes in the absorbance of the nucleic acid. These data show that *nef* sequences are normally present in a B-DNA conformation within the double-helix, and that a G-4 binder is able to induce their folding in G-4.

**Figure 5:**
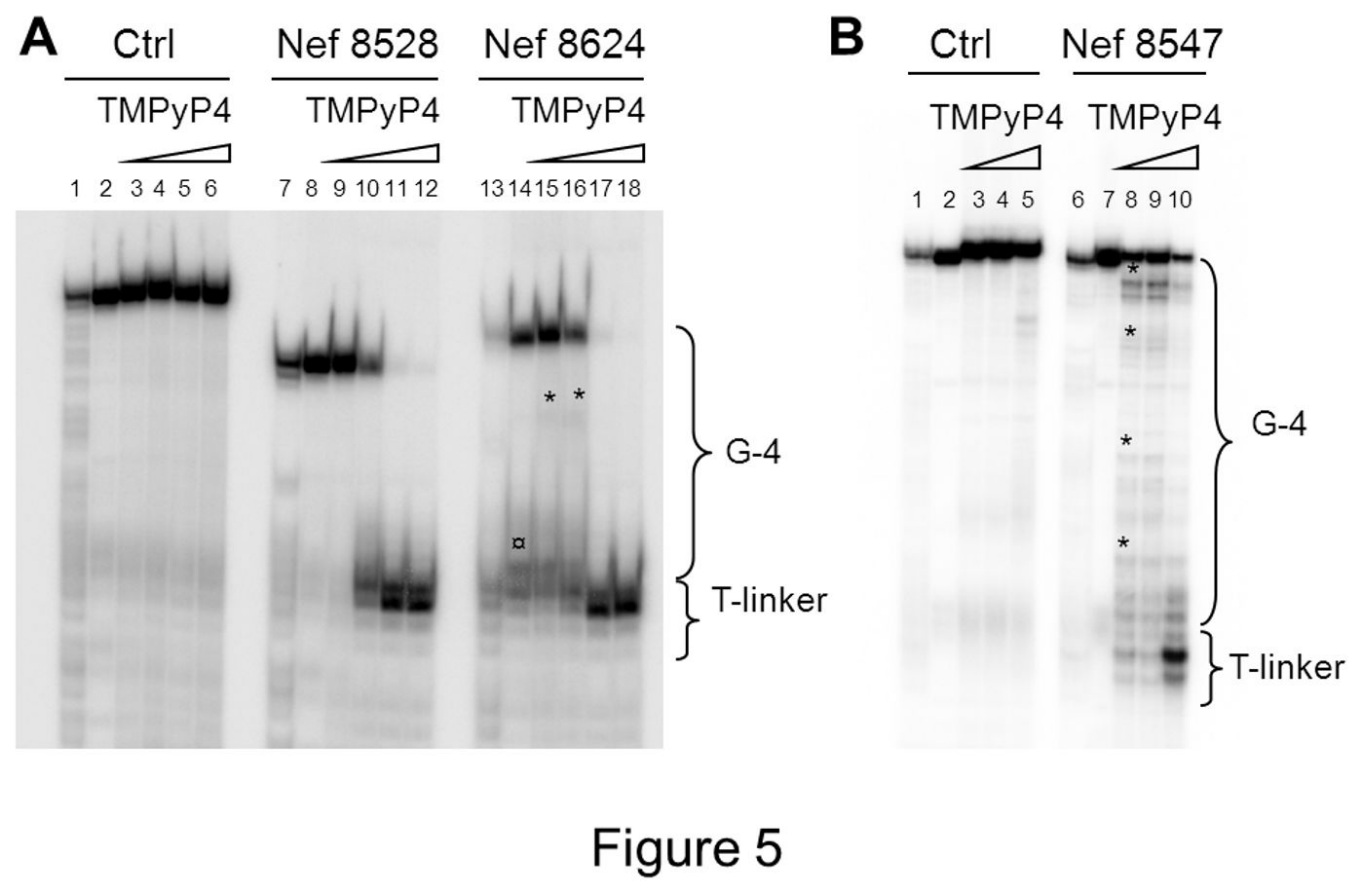
Taq polymerase stop assay. A) and B) Templates containing the G-4 nef sequences Nef8528, Nef8624 and Nef8547, a 4-T linker and a primer annealing region were allowed to fold and anneal to the P^32^-5’-end labelled primer in K^+^ 100 mM, treated with increasing concentrations of TMPyP4 (0-2 µM) and subjected to Taq polymerase extension. The control template contained a sequence unable to fold in G-4, and the same 4-T linker and primer annealing region as the nef templates. A) The * symbol indicates pausing sites in the G-4 region of nef templates. The ¤ symbol indicates a polymerase stop site obtained prior to addition of TMPyP4 in Nef8624. Lanes 1, 7 and 13 (A), and lanes 1 and 6 (B) were Maxam and Gilbert marker lanes performed on the double stranded PCR amplified region. Markers indicate the C-rich complementary strand.

G-4 folding in the selected sequences was additionally proved by the Taq polymerase stop assay. The three G-4 *nef* oligonucleotides were designed in order to contain additional flanking bases at both the 5’- and 3’-ends (see Materials and Methods Section); in particular, an additional sequence at their 3’-end was used as primer annealing region. A 4-T linker region was added to separate the 3’-end of the primer and the first G of the G-4 portion. An additional oligonucleotide lacking the possibility to fold in G-4 was designed and used as negative control. Primer annealing and G-4 folding were obtained by incubating the template G-4 forming oligonucleotides and the primer in K^+^ buffer at 95°C and slowly cooling down to room temperature. Taq polymerase was incubated with the different template/primer combinations in the presence of increasing amount of TMPyP4. As shown in Figure 5A, in the presence of both Nef8528 and Nef8624 templates, increasing drug concentrations induced arrest of the DNA polymerase processing at the T linker region, just before the G-4 folded region (lanes 10-12 and 17-18, Figure 5A), while no effect was detected in the negative control (lanes 2-6, Figure 5A). In the case of the Nef8624 template, at low amounts of TMPyP4, a polymerase pausing site was observed corresponding to the second G-tract of the Nef8624 oligonucleotide (* symbol, lanes 15-16, Figure 5A). In addition the polymerase was partially inhibited also in the absence of TMPyP4 (¤ symbol, lane 14, Figure 5A). We ascribed this behaviour to the fact that the G-4 conformation of Nef8624 was inherently very stable in the presence of K^+^ (T_m_ 59.8°C, see Table 3) and thus could affect polymerase activity even without G-4 ligands, similarly to other reported G-4 structures [54]. In the case of the Nef8547 template, several stop sites were observed in the G-4 forming template, while the polymerase was not inhibited in the control (compare lanes 2-5 and 7-10, Figure 5B). In particular, stop sites were clustered at G bases at low TMPyP4 concentrations (* symbols, lane 8, Figure 5B) and at the T-linker at higher G-ligand amounts (lane 10, Figure 5B).

Overall these data indicate that a G-4 binder can induce and stabilize the G-4 conformations at the *nef* DNA level.

### Stabilization of *nef* G-4 sequences by G-4 ligands affects gene expression

To check the effect of G-4 stabilizing ligands on gene expression, a GFP-based reporter gene assay was set up. The *nef* gene was cloned upstream of a GFP coding region in a plasmid optimized for protein expression in mammalian cells. In principle, G-4 folding in the *nef* sequence should impair the polymerase activity on the DNA template, therefore reducing expression of the fused Nef-GFP protein. Transfected cells were treated with TMPyP4 (10 µM) or a control compound, TMPyP2, a structural analogue of TMPyP4 which is not able to bind G-4s [55]; both compounds did not show toxicity on HEK293T cells up to 100 µM (data not shown). GFP fluorescence was quantified by flow cytometry-based analysis.

**Figure 6:**
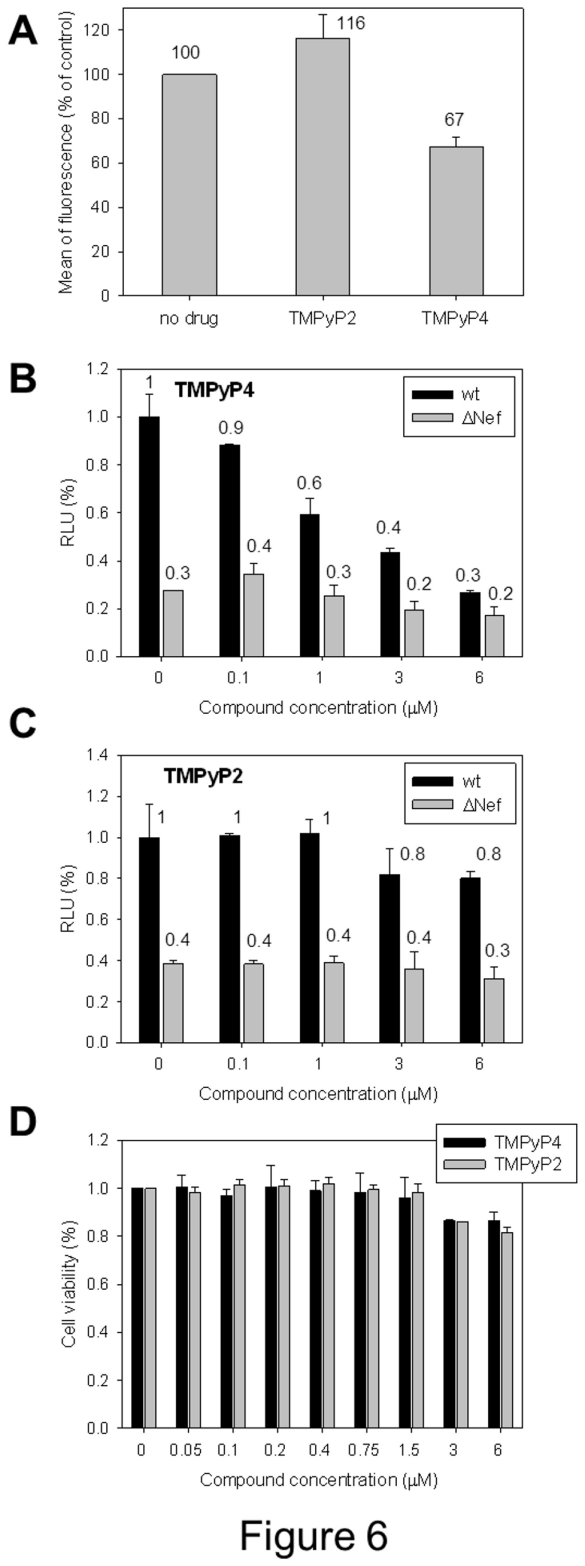
Effects of the stabilization of the *nef* G-4s by TMPyP4 on gene expression and viral infectivity in Nef sensitive cells. A) Effect of TMPyP4 and TMPyP2 on Nef-GFP expression measured by flow-cytometry. HEK 293T cells were transfected with a Nef-GFP encoding plasmid and treated with TMPyP4 or TMPyP2 (10 µM) for 24 h. Results are shown as percent mean of fluorescence relative to the control cells incubated ± SD (n = 4). Statistical difference was observed for TMPyP4 (p<0.05), but not for TMPyP2. B) and C) TZM-bl cells were infected with wild-type (black bars) and ΔNef (grey bars) HIV NL4-3 in the presence of either the G-4 ligand, TMPyP4 (B), or the negative control compound, TMPyP2 (C). After 48 h, infectivity was assessed as relative luciferase activity in infected cells. Results are shown as percent infectivity relative to the control cells incubated with carrier solvent (DMSO) ± SEM (n = 3). In B), no statistical difference was observed across ΔNef infected cells, even at the highest concentration (p > 0.15). In C), no statistical difference was observed for the wild-type virus at 3 µM and 6 µM (p >0.345 and >0.325, respectively) relative to the untreated control. The negative control compound, TMPyP2, further had no impact on the ΔNef virus at any concentration tested (p>0.29 at 6 µM). D) TZM-bl cell viability in the presence of compounds was assessed via the Cell-Titer Blue assay (Promega). TZM-bl cells were incubated with the indicated concentrations of compounds for 48 h and cell viability was assessed via the Cell-Titer Blue assay relative to control cells incubated with carrier solvent. Assays were done in triplicate.

As shown in Figure 6A, mean of fluorescence consistently decreased in the presence of TMPyP4, while slightly increased upon treatment with the control TMPyP2 indicating a selective impairment of gene expression mediated by the interaction of the G-4 ligand with the *nef* sequence.

### Effect of *nef* G-4 binding compounds on HIV-1 infectivity

Having observed an impairment of Nef protein expression levels, the effect of G-4 stabilizing ligands was next assessed in HIV-1 infected cells. As Nef has been previously shown to enhance HIV-1 infectivity [31,33,56,57], we evaluated the impact of TMPyP4 on infectivity using the TZM-bl reporter cell line [40,41]. TZM-bl cells support HIV-1 replication in a Nef-dependent manner and contain a luciferase reporter driven by the HIV-1 LTR in response to infection with HIV-1 [41,42]. As shown in Figure 6B, TMPyP4 impaired the enhancement of Nef-mediated viral infectivity in a dose-dependent manner, while the negative control (TMPyP2) had no effect (Figure 6C). In addition, a Nef-deleted virus (ΔNef) showed no significant effects by treatment with either TMPyP4 or TMPyP2 at the highest concentrations (Figure 6B-C). Cytoxicity assays further confirmed the observed impairment of viral infectivity as minimal toxicity was observed over the range of concentrations tested (0.1-6 µM) (Figure 6D). Taken together, these results provide support for a G-4 mediated, Nef-directed anti-retroviral mechanism of action.

## Discussion

To our knowledge, this is the first report indicating a) the presence of conserved G-rich sequences that can fold in the G-4 conformation within the HIV-1 proviral genome, b) the important role played by G-4 stabilization in both polymerase and viral infectivity inhibition.

The Nef protein has a fundamental function *in* ** *vivo* and its lack of activity (observed in Nef mutant HIV strains) prevents progression to the clinical development of AIDS [34]; therefore its depletion could have critical antiviral effects.

Here we demonstrated that in the HIV-1 Nef coding region three G-rich/G-4 prone tracts were closely clustered and located on both the leading and lagging strands where G-4-mediated induction of genetic instability and arrest of RNA-dependent polymerase have been described [11,13]. In addition, in a supercoiled environment, i-motif conformations may arise in the C-rich complementary sequences independently of G-4 formation and pH [1,58]. We thus foresaw the possibility of creating a structured environment upon induction of non-canonical nucleic acid conformations, with the possibility of blocking enzymes involved in Nef protein expression. The identified G-4 forming sequences comprised at least four tracts of two consecutive Gs, with the possibility to form G-4s with two stacked tetrads. Although in principle two G-tetrads would confer less stability than more extended G-tetrads, the existence and biological role of G-4s with two stacked tetrads has been reported in several cases [59–63]. Interestingly, in certain instances, two-tetrads G-4s showed higher stability than three-tetrads G-4s [64–67].

Upon analysis of all HIV-1 strains available in databases, we demonstrated that the G-rich region in the *nef* coding gene, and in particular the G pattern necessary for G-4 folding, is conserved within the HIV-1 M group and in its subtypes largely distributed worldwide. This information suggests that G-4 structures may form at this level and may also be involved in important viral functions. If this was the case, we argued that stabilization induced by G-4 binders would exert an effect observable at the viral level. All three putative G-4 *nef* sequences were able to fold in a G-4 conformation in the presence of G-4 binders. In most cases G-4 ligands varied the initial G-4 topology and augmented the T_m_ during temperature unfolding experiments, indicating an effective interaction between them and the G-4 *nef* oligonucleotides. Importantly, all G-4 *nef* sequences were able to form G-4 structures even in the presence of their complementary oligonucleotides. In the case of Nef8528 and Nef8547, they both existed in a fully double-stranded state and G-4 folding was induced upon incubation with the G-4 ligand. Conversely, Nef8624 was present in a partial G-4 topology even in the absence of the G-4 binder, as attested by both CD analysis and partial inhibition of the DNA polymerase activity prior to G-4 compound addition. This is in accordance with the higher T_m_ measured for the G-4 structure of Nef8624.

Most importantly, the ability to induce G-4 folding with consequent inhibition of viral infectivity was shown in HIV-1 infected cells. Within the cellular context of viral infection, the G-rich sequences are embedded in a double stranded state and yet small amounts of G-4 ligand were sufficient to impair the Nef-mediated enhancement of HIV-1 infectivity. The significant effect observed on the wt HIV-1 virus in Nef sensitive cells [45] and the negligible effect on the ΔNef virus further indicate an efficient inhibition of Nef protein expression.

Recent research on G-4 has revealed strong stabilization of tetraplex structures at the RNA level [68,69]. It is thus possible that, besides the DNA, the *nef* mRNA is also stabilized by G-4 compounds, therefore enhancing inhibition of Nef expression in infected cells. The fact that Nef8624, the most stable G-4 sequence, was able to pause the polymerase independently of the presence of G-4 ligands, suggests that it may naturally function as a molecular switch to assist protein expression or other unanticipated viral functions.

Moreover, G-4 stabilization within the *nef* coding region of the viral genome may impair not only Nef expression through inhibition of transcription that directly generates mRNAs, but also overall transcription for production of new copies of the RNA genome to be assembled in the viral progeny.

An additional point to be considered is that available G-4 ligands were developed principally against the telomeric G-4, and were shown to kill tumour cells through telomere dysfunction and telomerase inhibition [70,71]. G-4 stabilizing compounds interact with the G-4 structure both at the aromatic G-quartet level, which is common to all G-4 conformations, and at the loops, which conversely are unique to each G-4 folding [72]. Here we have shown that the G-4 ligand TMPyP4 is effective towards the virus with minimal effect on cell viability. Therefore a therapeutic window can be envisaged that would consent the employment of known G-4 binders as anti-HIV compounds. In addition, rational design of new G-4 ligands directed towards the tested G-4 *nef* sequences would likely augment the selective effects towards the virus. Detailed structural characterization of the G-4 *nef* sequences will be the first step to allow molecular modelling of new G-4 binding leads.

The results presented here may open a new avenue in the development of antiviral compounds with an unprecedented mechanism of action.

## References

[1] Brooks TA, Kendrick S, Hurley L (2010) Making sense of G-quadruplex and i-motif functions in oncogene promoters. FEBS J 277: 3459-3469. doi:10.1111/j.1742-4658.2010.07759.x. PubMed: 20670278. 20670278 10.1111/j.1742-4658.2010.07759.x PMC2971675

[2] Paeschke K, Simonsson T, Postberg J, Rhodes D, Lipps HJ (2005) Telomere end-binding proteins control the formation of G-quadruplex DNA structures in vivo. Nat Struct Mol Biol 12: 847-854. doi:10.1038/nsmb982. PubMed: 16142245. 16142245 10.1038/nsmb982

[3] Folini M, Venturini L, Cimino-Reale G, Zaffaroni N (2011) Telomeres as targets for anticancer therapies. Expert Opin Ther Targets 15: 579-593. PubMed: 21288186. 21288186 10.1517/14728222.2011.556621

[4] Huppert JL, Balasubramanian S (2007) G-quadruplexes in promoters throughout the human genome. Nucleic Acids Res 35: 406-413. PubMed: 17169996. 17169996 10.1093/nar/gkl1057 PMC1802602

[5] Balasubramanian S, Hurley LH, Neidle S (2011) Targeting G-quadruplexes in gene promoters: a novel anticancer strategy? Nat Rev Drug Discov 10: 261-275. doi:10.1038/nrd3428. PubMed: 21455236. 21455236 10.1038/nrd3428 PMC3119469

[6] Weitzmann MN, Woodford KJ, Usdin K (1997) DNA secondary structures and the evolution of hypervariable tandem arrays. J Biol Chem 272: 9517-9523. doi:10.1074/jbc.272.14.9517. PubMed: 9083093. 9083093 10.1074/jbc.272.14.9517

[7] Dunnick W, Hertz GZ, Scappino L, Gritzmacher C (1993) DNA sequences at immunoglobulin switch region recombination sites. Nucleic Acids Res 21: 365-372. doi:10.1093/nar/21.3.365. PubMed: 8441648. 8441648 10.1093/nar/21.3.365 PMC309126

[8] Hanakahi LA, Sun H, Maizels N (1999) High affinity interactions of nucleolin with G-G-paired rDNA. J Biol Chem 274: 15908-15912. doi:10.1074/jbc.274.22.15908. PubMed: 10336496. 10336496 10.1074/jbc.274.22.15908

[9] Law MJ, Lower KM, Voon HP, Hughes JR, Garrick D et al. (2011) ATR-X syndrome protein targets tandem repeats and influences allele-specific expression in a size-dependent manner. Cell 143: 367-378. 10.1016/j.cell.2010.09.023 21029860

[10] Maizels N (2006) Dynamic roles for G4 DNA in the biology of eukaryotic cells. Nat Struct Mol Biol 13: 1055-1059. doi:10.1038/nsmb1171. PubMed: 17146462. 17146462 10.1038/nsmb1171

[11] Lopes J, Piazza A, Bermejo R, Kriegsman B, Colosio A et al. (2011) G-quadruplex-induced instability during leading-strand replication. EMBO J 30: 4033-4046. doi:10.1038/emboj.2011.316. PubMed: 21873979. 21873979 10.1038/emboj.2011.316 PMC3209785

[12] Nambiar M, Goldsmith G, Moorthy BT, Lieber MR, Joshi MV et al. (2010) Formation of a G-quadruplex at the BCL2 major breakpoint region of the t(14;18) translocation in follicular lymphoma. Nucleic Acids Res 39: 936-948. PubMed: 20880994. 20880994 10.1093/nar/gkq824 PMC3035451

[13] Belotserkovskii BP, Liu R, Tornaletti S, Krasilnikova MM, Mirkin SM et al. (2010) Mechanisms and implications of transcription blockage by guanine-rich DNA sequences. Proc Natl Acad Sci U S A 107: 12816-12821. doi:10.1073/pnas.1007580107. PubMed: 20616059. 20616059 10.1073/pnas.1007580107 PMC2919923

[14] Duquette ML, Handa P, Vincent JA, Taylor AF, Maizels N (2004) Intracellular transcription of G-rich DNAs induces formation of G-loops, novel structures containing G4 DNA. Genes Dev 18: 1618-1629. doi:10.1101/gad.1200804. PubMed: 15231739. 15231739 10.1101/gad.1200804 PMC443523

[15] Baral A, Kumar P, Halder R, Mani P, Yadav VK et al. (2012) Quadruplex-single nucleotide polymorphisms (Quad-SNP) influence gene expression difference among individuals. Nucleic Acids Res 40: 3800-3811. doi:10.1093/nar/gkr1258. PubMed: 22238381. 22238381 10.1093/nar/gkr1258 PMC3351168

[16] Biffi G, Tannahill D, McCafferty J, Balasubramanian S (2013) Quantitative visualization of DNA G-quadruplex structures in human cells. Nat Chem 5: 182-186. doi:10.1038/nchem 1548 PubMed : 23422559 10.1038/nchem.1548 PMC3622242 23422559

[17] Ou TM, Lu YJ, Tan JH, Huang ZS, Wong KY et al. (2008) G-quadruplexes: targets in anticancer drug design. Chemmedchem 3: 690-713. doi:10.1002/cmdc.200700300. PubMed: 18236491. 18236491 10.1002/cmdc.200700300

[18] Fedoroff OY, Salazar M, Han H, Chemeris VV, Kerwin SM et al. (1998) NMR-Based model of a telomerase-inhibiting compound bound to G-quadruplex DNA. Biochemistry 37: 12367-12374. doi:10.1021/bi981330n. PubMed: 9730808. 9730808 10.1021/bi981330n

[19] Izbicka E, Wheelhouse RT, Raymond E, Davidson KK, Lawrence RA et al. (1999) Effects of cationic porphyrins as G-quadruplex interactive agents in human tumor cells. Cancer Res 59: 639-644. PubMed: 9973212. 9973212

[20] Campbell NH, Parkinson GN, Reszka AP, Neidle S (2008) Structural basis of DNA quadruplex recognition by an acridine drug. J Am Chem Soc 130: 6722-6724. doi:10.1021/ja8016973. PubMed: 18457389. 18457389 10.1021/ja8016973

[21] Kim MY, Vankayalapati H, Shin-Ya K, Wierzba K, Hurley LH (2002) Telomestatin, a potent telomerase inhibitor that interacts quite specifically with the human telomeric intramolecular g-quadruplex. J Am Chem Soc 124: 2098-2099. doi:10.1021/ja017308q. PubMed: 11878947. 11878947 10.1021/ja017308q

[22] Drygin D, Siddiqui-Jain A, O’Brien S, Schwaebe M, Lin A et al. (2009) Anticancer activity of CX-3543: a direct inhibitor of rRNA biogenesis. Cancer Res 69: 7653-7661. doi:10.1158/0008-5472.CAN-09-1304. PubMed: 19738048. 19738048 10.1158/0008-5472.CAN-09-1304

[23] Wieland M, Hartig JS (2009) Investigation of mRNA quadruplex formation in Escherichia coli. Nat Protoc 4: 1632-1640. doi:10.1038/nprot.2009.111. PubMed: 19876023. 19876023 10.1038/nprot.2009.111

[24] Beaume N, Pathak R, Yadav VK, Kota S, Misra HS et al. (2013) Genome-wide study predicts promoter-G4 DNA motifs regulate selective functions in bacteria: radioresistance of D. radiodurans involves G4 DNA-mediated regulation. Nucleic Acids Res 41: 76-89. doi:10.1093/nar/gkt004. PubMed: 23161683. 23161683 10.1093/nar/gks1071 PMC3592403

[25] Rawal P, Kummarasetti VB, Ravindran J, Kumar N, Halder K et al. (2006) Genome-wide prediction of G4 DNA as regulatory motifs: role in Escherichia coli global regulation. Genome Res 16: 644-655. doi:10.1101/gr.4508806. PubMed: 16651665. 16651665 10.1101/gr.4508806 PMC1457047

[26] Gallo RC, Montagnier L (2003) The discovery of HIV as the cause of AIDS. N Engl J Med 349: 2283-2285. doi:10.1056/NEJMp038194. PubMed: 14668451. 14668451 10.1056/NEJMp038194

[27] AIDSinfo (2011) HIV and Its Treatment- FDA-Approved Anti-HIV Medications. In: Services DoHaH, editor: U.S. Department of Health and Human Services ’ Guidelines.

[28] Hemelaar J, Gouws E, Ghys PD, Osmanov S (2011) Global trends in molecular epidemiology of HIV-1 during 2000-2007. AIDS 25: 679-689. doi:10.1097/QAD.0b013e328342ff93. PubMed: 21297424. 21297424 10.1097/QAD.0b013e328342ff93 PMC3755761

[29] Kuiken C, Leitner T, Foley B, Hahn B, Marx P et al. (2008) HIV Sequence Compendium 2008. Los Alamos National Laboratory, Theoretical Biology and Biophysics, Los Alamos, New Mexico. LaUR: 08-03719.

[30] Miller MD, Warmerdam MT, Gaston I, Greene WC, Feinberg MB (1994) The human immunodeficiency virus-1 nef gene product: a positive factor for viral infection and replication in primary lymphocytes and macrophages. J Exp Med 179: 101-113. doi:10.1084/jem.179.1.101. PubMed: 8270859. 8270859 10.1084/jem.179.1.101 PMC2191317

[31] Spina CA, Kwoh TJ, Chowers MY, Guatelli JC, Richman DD (1994) The importance of nef in the induction of human immunodeficiency virus type 1 replication from primary quiescent CD4 lymphocytes. J Exp Med 179: 115-123. doi:10.1084/jem.179.1.115. PubMed: 7903679. 7903679 10.1084/jem.179.1.115 PMC2191324

[32] Harris M (1999) HIV: a new role for Nef in the spread of HIV. Curr Biol 9: R459-R461. doi:10.1016/S0960-9822(99)80282-6. PubMed: 10375524. 10375524 10.1016/s0960-9822(99)80282-6

[33] Aiken C, Trono D (1995) Nef stimulates human immunodeficiency virus type 1 proviral DNA synthesis. J Virol 69: 5048-5056. PubMed: 7541845. 7541845 10.1128/jvi.69.8.5048-5056.1995 PMC189322

[34] Salvi R, Garbuglia AR, Di Caro A, Pulciani S, Montella F et al. (1998) Grossly defective nef gene sequences in a human immunodeficiency virus type 1-seropositive long-term nonprogressor. J Virol 72: 3646-3657. PubMed: 9557645. 9557645 10.1128/jvi.72.5.3646-3657.1998 PMC109585

[35] Kestler HW 3rd, Ringler DJ, Mori K, Panicali DL, Sehgal PK et al. (1991) Importance of the nef gene for maintenance of high virus loads and for development of AIDS. Cell 65: 651-662. doi:10.1016/0092-8674(91)90097-I. PubMed: 2032289. 2032289 10.1016/0092-8674(91)90097-i

[36] Richter SN, Frasson I, Palù G (2009) Strategies for inhibiting function of HIV-1 accessory proteins: a necessary route to AIDS therapy? Curr Med Chem 16: 267-286. doi:10.2174/092986709787002646. PubMed: 19149577. 19149577 10.2174/092986709787002646

[37] Lyonnais S, Gorelick RJ, Mergny JL, Le Cam E, Mirambeau G (2003) G-quartets direct assembly of HIV-1 nucleocapsid protein along single-stranded DNA. Nucleic Acids Res 31: 5754-5763. doi:10.1093/nar/gkg716. PubMed: 14500839. 14500839 10.1093/nar/gkg716 PMC206446

[38] Sundquist WI, Heaphy S (1993) Evidence for interstrand quadruplex formation in the dimerization of human immunodeficiency virus 1 genomic RNA. Proc Natl Acad Sci U S A 90: 3393-3397. doi:10.1073/pnas.90.8.3393. PubMed: 8475087. 8475087 10.1073/pnas.90.8.3393 PMC46306

[39] Kikin O, D’Antonio L, Bagga PS (2006) QGRS Mapper: a web-based server for predicting G-quadruplexes in nucleotide sequences. Nucleic Acids Res 34: W676-W682. doi:10.1093/nar/gkj467. PubMed: 16845096. 16845096 10.1093/nar/gkl253 PMC1538864

[40] Platt EJ, Wehrly K, Kuhmann SE, Chesebro B, Kabat D (1998) Effects of CCR5 and CD4 cell surface concentrations on infections by macrophagetropic isolates of human immunodeficiency virus type 1. J Virol 72: 2855-2864. PubMed: 9525605. 9525605 10.1128/jvi.72.4.2855-2864.1998 PMC109730

[41] Derdeyn CA, Decker JM, Sfakianos JN, Wu X, O’Brien WA et al. (2000) Sensitivity of human immunodeficiency virus type 1 to the fusion inhibitor T-20 is modulated by coreceptor specificity defined by the V3 loop of gp120. J Virol 74: 8358-8367. doi:10.1128/JVI.74.18.8358-8367.2000. PubMed: 10954535. 10954535 10.1128/jvi.74.18.8358-8367.2000 PMC116346

[42] Emert-Sedlak L, Kodama T, Lerner EC, Dai W, Foster C et al. (2009) Chemical library screens targeting an HIV-1 accessory factor/host cell kinase complex identify novel antiretroviral compounds. ACS Chem Biol 4: 939-947. doi:10.1021/cb900195c. PubMed: 19807124. 19807124 10.1021/cb900195c PMC2861989

[43] Narute PS, Smithgall TE (2012) Nef alleles from all major HIV-1 clades activate Src-family kinases and enhance HIV-1 replication in an inhibitor-sensitive manner. PLOS ONE 7: e32561. doi:10.1371/journal.pone.0032561. PubMed: 22393415. 22393415 10.1371/journal.pone.0032561 PMC3290594

[44] Poe JA, Smithgall TE (2009) HIV-1 Nef dimerization is required for Nef-mediated receptor downregulation and viral replication. J Mol Biol 394: 329-342. doi:10.1016/j.jmb.2009.09.047. PubMed: 19781555. 19781555 10.1016/j.jmb.2009.09.047 PMC2783173

[45] Emert-Sedlak LA, Narute P, Shu ST, Poe JA, Shi H et al. (2013) Effector kinase coupling enables high-throughput screens for direct HIV-1 Nef antagonists with antiretroviral activity. Chem Biol 20: 82-91. doi:10.1016/j.chembiol.2012.11.005. PubMed: 23352142. 23352142 10.1016/j.chembiol.2012.11.005 PMC3559019

[46] Grzesiek S, Bax A, Clore GM, Gronenborn AM, Hu JS et al. (1996) The solution structure of HIV-1 Nef reveals an unexpected fold and permits delineation of the binding surface for the SH3 domain of Hck tyrosine protein kinase. Nat Struct Biol 3: 340-345. doi:10.1038/nsb0496-340. PubMed: 8599760. 8599760 10.1038/nsb0496-340

[47] Lee CH, Saksela K, Mirza UA, Chait BT, Kuriyan J (1996) Crystal structure of the conserved core of HIV-1 Nef complexed with a Src family SH3 domain. Cell 85: 931-942. doi:10.1016/S0092-8674(00)81276-3. PubMed: 8681387. 8681387 10.1016/s0092-8674(00)81276-3

[48] Cheng H, Hoxie JP, Parks WP (1999) The conserved core of human immunodeficiency virus type 1 Nef is essential for association with Lck and for enhanced viral replication in T-lymphocytes. Virology 264: 5-15. doi:10.1006/viro.1999.9937. PubMed: 10544125. 10544125 10.1006/viro.1999.9937

[49] Bouaziz S, Kettani A, Patel DJ (1998) A K cation-induced conformational switch within a loop spanning segment of a DNA quadruplex containing G-G-G-C repeats. J Mol Biol 282: 637-652. doi:10.1006/jmbi.1998.2031. PubMed: 9737927. 9737927 10.1006/jmbi.1998.2031

[50] Vorlickova M, Kejnovska I, Sagi J, Renciuk D, Bednarova K et al. (2012) Circular dichroism and guanine quadruplexes. Methods. 10.1016/j.ymeth.2012.03.011 22450044

[51] Guédin A, Gros J, Alberti P, Mergny JL (2010) How long is too long? Effects of loop size on G-quadruplex stability. Nucleic Acids Res 38: 7858-7868. doi:10.1093/nar/gkq639. PubMed: 20660477. 20660477 10.1093/nar/gkq639 PMC2995061

[52] Kypr J, Kejnovská I, Renciuk D, Vorlícková M (2009) Circular dichroism and conformational polymorphism of DNA. Nucleic Acids Res 37: 1713-1725. doi:10.1093/nar/gkp026. PubMed: 19190094. 19190094 10.1093/nar/gkp026 PMC2665218

[53] Morris MJ, Wingate KL, Silwal J, Leeper TC, Basu S (2012) The porphyrin TmPyP4 unfolds the extremely stable G-quadruplex in MT3-MMP mRNA and alleviates its repressive effect to enhance translation in eukaryotic cells. Nucleic Acids Res, 40: 4137–45. PubMed: 22266651. 22266651 10.1093/nar/gkr1308 PMC3351169

[54] Palumbo SL, Ebbinghaus SW, Hurley LH (2009) Formation of a unique end-to-end stacked pair of G-quadruplexes in the hTERT core promoter with implications for inhibition of telomerase by G-quadruplex-interactive ligands. J Am Chem Soc 131: 10878-10891. doi:10.1021/ja902281d. PubMed: 19601575. 19601575 10.1021/ja902281d PMC2761083

[55] Han H, Langley DR, Rangan A, Hurley LH (2001) Selective interactions of cationic porphyrins with G-quadruplex structures. J Am Chem Soc 123: 8902-8913. doi:10.1021/ja002179j. PubMed: 11552797. 11552797 10.1021/ja002179j

[56] Chowers MY, Spina CA, Kwoh TJ, Fitch NJ, Richman DD et al. (1994) Optimal infectivity in vitro of human immunodeficiency virus type 1 requires an intact nef gene. J Virol 68: 2906-2914. PubMed: 8151761. 8151761 10.1128/jvi.68.5.2906-2914.1994 PMC236779

[57] Vermeire J, Vanbillemont G, Witkowski W, Verhasselt B (2011) The Nef-infectivity enigma: mechanisms of enhanced lentiviral infection. Curr HIV Res 9: 474-489. doi:10.2174/157016211798842099. PubMed: 22103831. 22103831 10.2174/157016211798842099 PMC3355465

[58] Sun D, Hurley LH (2009) The importance of negative superhelicity in inducing the formation of G-quadruplex and i-motif structures in the c-Myc promoter: implications for drug targeting and control of gene expression. J Med Chem 52: 2863-2874. doi:10.1021/jm900055s. PubMed: 19385599. 19385599 10.1021/jm900055s PMC2757002

[59] Raiber EA, Kranaster R, Lam E, Nikan M, Balasubramanian S (2012) A non-canonical DNA structure is a binding motif for the transcription factor SP1 in vitro. Nucleic Acids Res 40: 1499-1508. doi:10.1093/nar/gkr882. PubMed: 22021377. 22021377 10.1093/nar/gkr882 PMC3287196

[60] Basundra R, Kumar A, Amrane S, Verma A, Phan AT et al. (2010) A novel G-quadruplex motif modulates promoter activity of human thymidine kinase 1. FEBS J 277: 4254-4264. doi:10.1111/j.1742-4658.2010.07814.x. PubMed: 20849417. 20849417 10.1111/j.1742-4658.2010.07814.x

[61] Jang MY, Yarborough OH 3rd, Conyers GB, McPhie P, Owens RA (2005) Stable secondary structure near the nicking site for adeno-associated virus type 2 Rep proteins on human chromosome 19. J Virol 79: 3544-3556. doi:10.1128/JVI.79.6.3544-3556.2005. PubMed: 15731249. 15731249 10.1128/JVI.79.6.3544-3556.2005 PMC1075745

[62] Kettani A, Bouaziz S, Wang W, Jones RA, Patel DJ (1997) Bombyx mori single repeat telomeric DNA sequence forms a G-quadruplex capped by base triads. Nat Struct Biol 4: 382-389. doi:10.1038/nsb0597-382. PubMed: 9145109. 9145109 10.1038/nsb0597-382

[63] Xu Y, Sugiyama H (2006) Formation of the G-quadruplex and i-motif structures in retinoblastoma susceptibility genes (Rb). Nucleic Acids Res 34: 949-954. doi:10.1093/nar/gkj485. PubMed: 16464825. 16464825 10.1093/nar/gkj485 PMC1361614

[64] Hu L, Lim KW, Bouaziz S, Phan AT (2009) Giardia telomeric sequence d(TAGGG)4 forms two intramolecular G-quadruplexes in K+ solution: effect of loop length and sequence on the folding topology. J Am Chem Soc 131: 16824-16831. doi:10.1021/ja905611c. PubMed: 19874015. 19874015 10.1021/ja905611c

[65] Lim KW, Alberti P, Guédin A, Lacroix L, Riou JF et al. (2009) Sequence variant ( CTAGGG)n in the human telomere favors a G-quadruplex structure containing a G.C.G.C tetrad. Nucleic Acids Res 37: 6239-6248. doi:10.1093/nar/gkp630. PubMed: 19692585. 19692585 10.1093/nar/gkp630 PMC2764449

[66] Lim KW, Amrane S, Bouaziz S, Xu W, Mu Y et al. (2009) Structure of the human telomere in K+ solution: a stable basket-type G-quadruplex with only two G-tetrad layers. J Am Chem Soc 131: 4301-4309. doi:10.1021/ja807503g. PubMed: 19271707. 19271707 10.1021/ja807503g PMC2662591

[67] Phan AT (2010) Human telomeric G-quadruplex: structures of DNA and RNA sequences. FEBS J 277: 1107-1117. doi:10.1111/j.1742-4658.2009.07464.x. PubMed: 19951353. 19951353 10.1111/j.1742-4658.2009.07464.x

[68] Mullen MA, Assmann SM, Bevilacqua PC (2012) Toward a digital gene response: RNA G-quadruplexes with fewer quartets fold with higher cooperativity. J Am Chem Soc 134: 812-815. doi:10.1021/ja2096255. PubMed: 22239732. 22239732 10.1021/ja2096255

[69] Zhang AY, Bugaut A, Balasubramanian S (2011) A sequence-independent analysis of the loop length dependence of intramolecular RNA G-quadruplex stability and topology. Biochemistry 50: 7251-7258. doi:10.1021/bi200805j. PubMed: 21744844. 21744844 10.1021/bi200805j PMC3522851

[70] Neidle S (2010) Human telomeric G-quadruplex: the current status of telomeric G-quadruplexes as therapeutic targets in human cancer. FEBS J 277: 1118-1125. doi:10.1111/j.1742-4658.2009.07463.x. PubMed: 19951354. 19951354 10.1111/j.1742-4658.2009.07463.x

[71] De Cian A, Lacroix L, Douarre C, Temime-Smaali N, Trentesaux C et al. (2008) Targeting telomeres and telomerase. Biochimie 90: 131-155. doi:10.1016/j.biochi.2007.07.011. PubMed: 17822826. 17822826 10.1016/j.biochi.2007.07.011

[72] Neidle S (2009) The structures of quadruplex nucleic acids and their drug complexes. Curr Opin Struct Biol 19: 239-250. doi:10.1016/j.sbi.2009.04.001. PubMed: 19487118. 19487118 10.1016/j.sbi.2009.04.001

[73] Geyer M, Fackler OT, Peterlin BM (2001) Structure--function relationships in HIV-1 Nef. EMBO Rep 2: 580-585. doi:10.1093/embo-reports/kve141. PubMed: 11463741. 11463741 10.1093/embo-reports/kve141 PMC1083955

